# The Relationship between Overweight/Obesity and Executive Control in College Students: The Mediating Effect of BDNF and 5-HT

**DOI:** 10.3390/life11040313

**Published:** 2021-04-03

**Authors:** Jing Si, Haidi Zhang, Lina Zhu, Aiguo Chen

**Affiliations:** 1 College of Physical Education, Yangzhou University, Yangzhou 225127, China; mx120180354@yzu.edu.cn (J.S.); mx120180351@yzu.edu.cn (H.Z.); 2 School of Physical Education and Sports Science, Beijing Normal University, Beijing 100875, China; zhulina827@mail.bnu.edu.cn

**Keywords:** overweight, obesity, executive control, 5-HT, BDNF

## Abstract

The main aim of this study was to explore the association between overweight/obesity and executive control (EC) in young adults, and to further analyze the mediating effect of brain-derived neurotrophic factor (BDNF) and serotonin (5-hydroxytryptamine (5-HT)) on the relationship between overweight/obesity and EC. A total of 449 college students aged between 18 and 20 years were recruited for the study between March and December 2019. Their height and weight were then measured professionally. Subsequently, body mass index (BMI) was calculated as weight (kg) divided by the square of height (m). The EC of the participants was then estimated using the Flanker task, while their serum BDNF levels and 5-HT levels were measured using an enzyme-linked immunosorbent assay (ELISA) kit. Finally, the multiple intermediary models in SPSS were used to analyze the mediating effect of 5-HT and BDNF between overweight/obesity and EC. The result show that the overweight/obesity of college students was positively correlated with the response of EC (*p* ≤ 0.005). However, it was negatively correlated with BDNF (*p* ≤ 0.05) and 5-HT (*p* ≤ 0.05). Moreover, BDNF (*p* ≤ 0.001) and 5-HT (*p* ≤ 0.001) were negatively correlated with the response of EC. The BDNF level played a partial mediating role between overweight/obesity and EC that accounted for 7.30% of the total effect value. Similarly, the 5-HT of college students played a partial mediating role between overweight/obesity and EC that accounted for 8.76% of the total effect value. Gender and age had no regulatory effect on the relationship between overweight/obesity, BDNF, 5-HT, and EC. This study provides the evidence that 5-HT and BDNF mediated the association between overweight/obesity and executive control. It is indicated that 5-HT and BDNF might be the biological pathways underpinning the link between overweight/obesity and executive control.

## 1. Introduction

By 2017, about 2.2 billion people were overweight (body mass index (BMI) ≥24) in the world, accounting for about one third of the global population, of whom about 712 million people (10% of the global population) were obese (BMI ≥28) people [1,2]. A recent study on the physical health of Chinese college students reported that the overweight and obesity rates were increasing yearly among the college students [3]. Remarkably, overweight/obesity in early adulthood are associated with higher risks of cardiovascular disease in late life [4,5], and it also negatively affects academic performance [6,7,8] and executive control (EC), becoming evident in accelerated cognitive decline and brain atrophy in later years [9,10]. Executive control plays a pivotal role in executive functions [11]. There are many neuropsychological paradigms can measure the performance of executive control including the Flanker task, Stroop task, and Go/No go task [12]. A previous study reported that poor development of EC leads to abnormal cognitive, emotional, and social functions in individuals, thereby adversely affecting their social achievements in adulthood [13]. Therefore, most researchers focus on the relationship between overweight/obesity and EC, with their findings suggesting that there are different mechanisms through which over-weight/obesity affects EC, and most of the research is related to the neural mechanism [14,15]. Recently, increasing attention has been focused on the biological pathway between the overweight/obesity on EC [16] and, as biogenic regulator factors, serotonin (5-hydroxytryptamine (5-HT)) and brain-derived neurotrophic factor (BDNF) have been studied extensively [17].

Within the brain 5-HT is widely distributed because it is an important neurotransmitter whose corresponding signal pathway participates in the physiological activities of the brain [18]. This makes it have a profound impact on EC [19,20,21]. It was additionally found that overweight/obesity can reduce 5-HT production. The disruption of the energy balance due to excess caloric intake and reduced energy consumption cause onset of metabolic disorders, leading to decreased 5-HT levels [22,23,24,25,26]. At the same time, many scholars use functional magnetic resonance imaging (fMRI) technology to show the acute influence that the drug regulating the 5-HT system has on brain activation in the process of individual participation in EC. Their results have shown that the regulation associated with EC is probably due to a large amount of serotonergic projection from the raphe nucleus to the basal ganglia, which clearly supports the influence of 5-HT on EC [27,28].

Based on the available scientific evidence, we proposed the first hypotheses: 5-HT can be used as an intermediary variable for the effect of overweight/obesity on EC.

Brain-derived neurotrophic factor (BDNF) is the richest neurotrophic factor in the body, which plays its role through binding to TrkB [29]. The cascade reaction between BDNF and the activation of TrkB, and its downstream signals is very important for cognitive function [30]. A previous study reported that a low level of BDNF is associated with cognitive impairment [31]. Notably, the improvement of EC ability is usually accompanied by the increase of BDNF levels [32,33]. Overweight/obesity not only affects 5-HT; it also affects BDNF, as suggested by the observation that people with obesity have low circulating levels of BDNF [34,35,36,37].

Based on the available scientific evidence, we proposed the second hypotheses: BDNF can be used as an intermediary variable for the effect of overweight/obesity on EC.

Accumulating evidence postulates that adolescents with higher BMI have a lower ability to suppress interference information unrelated to task [38]. They also have lower than normal levels of BDNF and 5-HT in peripheral blood [39,40]. Several previous studies have reported that an individual’s 5-HT and BDNF levels are affected by body fat [41,42], and the performance of the EC [43,44]. Along the same lines, overweight/obesity can directly affect the performance of the EC [45,46]. These reports suggest that the four factors are correlated with each other. It is worth noting that both 5-HT and BDNF belong to the molecular biological level, constituting common intracellular signaling pathways and transcription factors, BDNF control over the development and function of 5-HT, as well as 5-HT regulation of BDNF expression and signaling [47]. In this study, we hypothesized that the related 5-HT and BDNF levels play an intermediary role in the influence of overweight/obesity on EC.

Based on the available scientific evidence, we proposed the final hypotheses: Both 5-HT and BDNF are used as intermediary variables for the effect of overweight/obesity on EC to construct a multiple intermediary mode.

The present study, therefore, examined whether 5-HT and BDNF mediates the association between overweight/obesity and EC. Drawing on literature showing that overweight/obesity is associated with EC, and 5-HT and BDNF are related to overweight/obesity and EC, we hypothesized that 5-HT and BDNF would mediate the association between overweight/obesity and EC.

## 2. Materials and Methods

### 2.1. Participants

A total of 449 freshmen from 19–20 years old from the university with similar sociocultural environments and following a common prescribed syllabus and examination evaluation pattern were invited to participate in this study during the period of March and December 2019. Students with any acute or chronic health problems, such as diabetes, cardiovascular diseases, infections, asthma, malabsorption, physical disability, musculoskeletal disorders, mental disabilities, and/or concentration problems were excluded from the study. Also, they completed a set of questionnaires about the history of hereditary disease or misuse of drugs and general intelligence (Raven’s Standard Progressive Matrices, SPM). Parents (of the included students) and students were told the purpose of study and were required to give written informed consent prior to their joining the project. Our pooled dataset eventually included 449 samples. Table 1 outlines the demographic characteristics of these participants.

### 2.2. Somatometry Measurement

The height and weight of all participants were measured using a tape measure and an electronic scale. Overweight/obesity was then calculated by dividing the weight (in kilograms) by height (in meters squared). Overweight and obesity scores were based on the data obtained from a large-scale survey conducted on the Chinese population in 2003 which reported that the limited judging of overweight and obesity in Chinese adults was BMI ≥24 kg/m^2^ = overweight and BMI ≥28 kg/m^2^ = obesity [48]. Our results indicated that 50 boys were overweight and 26 were obese, while 39 girls were overweight and 13 were obese.

### 2.3. Flanker Task

The Flanker task is one of the most common procedures to assess EC. The executive control measurement tool developed by Chen et al. [49] was adopted and the measurement process completed on a computer. Furthermore, all the programs were realized using the E-prime1.1 system, while the flanker experimental paradigm was used for measurement. Based on the findings of peer experts, the task has proved to be highly reliable and valid in the measurement of college students’ EC. At the beginning of the experimental session, participants performed a practice trial of the flanker task to familiarize themselves with the experimental task and to ensure correct task performance. In addition, prior to the formal task, all participants practiced the trials multiple times until individual accuracy (percent correct) reached 80%. As a result, it was difficult to obtain a significant difference in accuracy. In early adulthood, cognitive abilities are firmly established, therefore, the consistent task was relatively easy for this age group. *The task is based on the theory that states: ‘the smaller the inconsistent reaction time (Inconsistent RT) indicate the better the ability of execution control”* [50]. The E-Prime program was used to display an arranged simulation task. All participants completed the Flanker task test in a quiet, spacious, and bright environment.

### 2.4. Brain-Derived Neurotrophic Factor (BDNF) and 5-Hydroxytryptamine (5-HT) Analysis

Blood samples were collected from the participants’ antecubital vein into a serum separator tube between 7.45 and 8.00 a.m. and left to stand for 30 min to clot. The blood was then centrifuged at 1000× *g* for 30 min in order to separate it into its components. Subsequently, serum samples were collected and aliquots stored at −80 °C awaiting analysis. According to the manufacturer’s instructions, BDNF levels and 5-HT were measured by enzyme-linked immunosorbent assay (ELISA) kit. The absorbance of the serum samples was then measured within 30 min using a microplate reader set at a wavelength of 450 nm in order to determine the concentration of 5-HT and BDNF based on a standard curve. All assays were performed in duplicates and the obtained mean values were used for subsequent analysis.

### 2.5. Statistical Analysis

All statistical analyses were done using the SPSS statistical software version 22.0 (SPSS Inc., Chicago, IL, USA). Data were presented as means, standard deviations, or relative frequencies (*n*, %). Means, standard deviations, and errors were calculated for overweight/obesity, EC, and serum 5-HT and BDNF levels for descriptive purposes. In addition, Pearson’s correlation coefficients were measured to examine the relationships among the serum 5-HT levels, BDNF levels, overweight/obesity, and the scores of the Flanker task for all the participants. Significance was defined as a *p*-value less than or equal to 0.05 for all inferential statistical analyses.

A mediation analysis was then performed using overweight/obesity as the predictor variable (X), and BDNF, 5-HT (M), and EC as the outcome variables (Y) in order to directly test the presence of an indirect effect of overweight/obesity on EC through BDNF and 5-HT. A bootstrapping approach, as implemented in the SPSS macro PROCESS, was applied (http://www.processmacro.org accessed on 2 March 2021) for this analysis step because it has been shown to provide reliable results in previous neuroimaging research [51,52,53]. In the bootstrapping approach, PROCESS was used to estimate the direct and indirect effects between defined sets of variables by applying an ordinary least squares path analytic framework. Inference of indirect (mediated) effects was subsequently assessed using bootstrap confidence intervals (CI). Significance of indirect effects was assumed if the 95% confidence interval (95%-*CI*) did not include a zero. The number of bootstrap samples was set to *n* = 5000, and unstandardized regression coefficients (*coeff*) and standard errors (*SE*) were presented for each effect. Mediation analyses were also repeated using standardized (z-trans formed) variables in order to obtain standardized regression coefficients (*Std coeff*). This was done to enhance comparability with the existing literatures. In line with all other analyses’ steps, the mediation analysis was first conducted without any covariates and then repeated using age and gender as nuisance regressors in the model.

### 2.6. Procedure

Volunteers were orally explained the venipuncture process, and then asked to eat a daily diet before the study and to refrain from drinking alcohol for 24 h prior to the study. Four mL of overnight fasting venous blood sample was then collected in the morning and used to determine the serotonin levels of the volunteers. The next day after blood was drawn, an anthropometric measurement was performed, and then the participant completed the Flanker task for 10 to 20 min.

## 3. Results

### 3.1. Descriptive Results, Demographics, and Physiological Variables

The descriptive characteristics of the participants are displayed in Table 1. The independent sample *t*-test was used to analyze differences in height, weight, BMI, 5-HT, BDNF, and EC performance of college students based on gender. The obtained results indicated that there were significant differences in weight (*p* ≤ 0.05, *Cohen’s d* = 1.01) between males and females. However, there were insignificant differences in overweight/obesity (*p* > 0.05, *Cohen’s d* = 0.22), EC (*p* > 0.05, *Cohen’s d* = −0.10), BDNF (*p* > 0.05, *Cohen’s d* = −0.20), and 5-HT (*p* > 0.05, *Cohen’s d* = 0.15) between the two genders (Table 1). Therefore, gender differences were not included in the analysis conducted in this study, and gender was not taken as a regulatory variable in the construction of multiple mediation models.

### 3.2. Behavioral Correlation Analysis of Overweight/Obesity, BDNF, 5-HT and Executive Control (EC) of College Students

**Figure 1:**
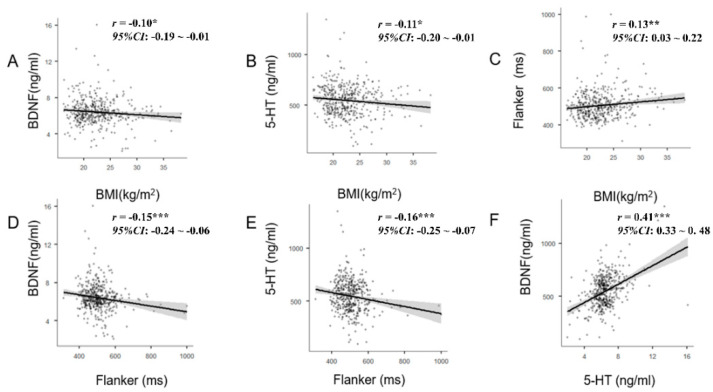
(**A**) BMI was negatively correlated with BDNF; (**B**) BMI was negatively correlated with 5-HT; (**C**) BMI was positively correlated with Flanker; (**D**) Flanker was negatively correlated with BDNF; (**E**) Flanker was negatively correlated with 5-HT; and (**F**) BDNF was positively correlated with 5-HT. 5-HT: Serotonin, BDNF: Brain-derived Neurotrophic Factor, BMI: Body Mass Index, Inconsistent RT: inconsistent reaction time. * Denotes statistical significance at *p* ≤ 0.05. ** Denotes statistical significance at *p* ≤ 0.01. *** Denotes statistical significance at *p* ≤ 0.001.

Behavioral correlation analysis between the variables was performed using Pearson correlation analysis (Figure 1). The overweight/obesity of college students was positively correlated with the response of EC (*r* = 0.13, *p* ≤ 0.01). However, it was negatively correlated with BDNF (*r* = −0.10, *p* ≤ 0.05) and 5-HT (*r* = −0.11, *p* ≤ 0.05). Along the same lines, BDNF (*r* = −0.15, *p* ≤ 0.001) and 5-HT (*r* = −0.16, *p* ≤ 0.001) were negatively correlated with the EC, while BDNF was positively correlated with 5-HT (*r* = 0.41, *p* ≤ 0.001).

### 3.3. Mediation Role of 5-HT and BDNF between Overweight/Obesity and Overweight/Obesity

The multiple mediation analysis showed that the overall impact of overweight/obesity on EC was significant (*B* = 0.14, *p* ≤ 0.05) (Table 2). In addition, the specific indirect effect mediators of X on Y are listed in Table 2. With respect to the specific direct effect of each proposed mediator between overweight/obesity and EC, 5-HT (*B* = −0.10, *p* ≤ 0.05) and BDNF (*B* = −0.11, *p* ≤ 0.05) were significant.

**Figure 2:**
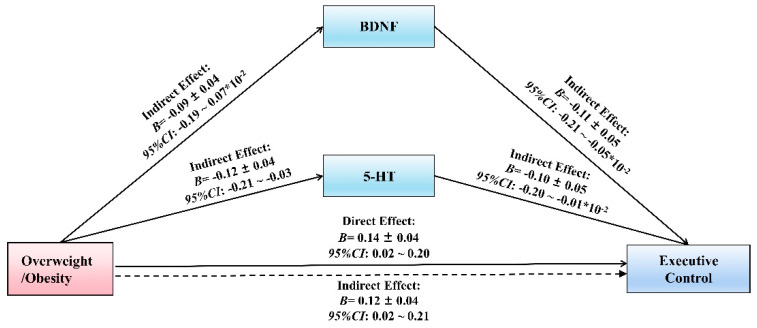
Mediation model of BDNF and 5-HT in the relationship between overweight/obesity and Executive Control. 5-HT: Serotonin, BDNF: Brain-derived Neurotrophic Factor. All variables in the model are substituted into the regression equation by the standardized variables.

Moreover, both the upper and lower bounds of the bootstrap 95% confidence interval of the direct effect of overweight/obesity on EC and BDNF’s mediating effect with 5-HT did not contain a 0 (Table 3; Figure 2). This indicated that overweight/obesity could directly predict the EC and also through BDNF’s mediating effect with 5-HT (Table 3). The results indicated that the direct effect of overweight/obesity accounted for 83.94%, the mediating effect of 5-HT accounted for 8.76%, and the mediating effect of BDNF accounted for 7.30%.

Further studies will be required to reveal the interrelationship between the 4 parameters (overweight/obesity, EC, 5-HT and BDNF), and the approach of Preacher and Hayes (2004) was used to test the saliency of the indirect effects [32]. In the model (Figure 2), 5-HT and BDNF were defined of the relationship between overweight/obesity and EC as mediators. A significant pathway between overweight/obesity and EC resulted.

## 4. Discussion

The results obtained in this study indicated that 5-HT and BDNF were significant mediators in the link between overweight/obesity and EC, which validated our hypothesis [54,55,56].

This study found that the higher the BMI, the lower the EC performance, indicating that obesity has a significant negative predictive effect on the EC of individuals with college students. The direct effect size was 83.94%. Previous studies on the relationship between obesity and duration of sedentary behaviors showed that obese people tend to have a longer sitting time and lower levels of aerobic fitness [57]. Meanwhile, the findings also indicate that lower aerobic fitness and sedentary behavior are related to inferior executive control [58,59]. Therefore, the damage of EC performance attributed to overweight/obesity can be counteracted by having high levels of aerobic fitness and changed sedentary behaviors of obese people [60,61].

This study further used PROCESS to establish the mediating effect test based on the correlation among overweight/obesity, 5-HT, and EC. The results indicated that the 5-HT levels of college students played a partial mediating role between over-weight/obesity and EC. First, overweight/obesity probably result in lower 5-HT levels [26] and, thus diminished the core subcortical/cortical region activity such as amygdala and medial prefrontal cortex, and reduce the activity of the underlying neural networks, which might impair the performance of EC [62,63,64]. Second, high 5-HT levels can exert direct effects on their receptors within the prefrontal cortex and hippocampus, and improve performance of EC at least in part via those mechanisms [65]. The results showed that serum 5-HT levels were responsible for 8.76% of the overweight/obesity associated with EC. This indicated that overweight/obesity affects EC severity through the 5-HT levels, thus, both these results and ours provide preliminary evidence in support of the idea that overweight/obesity-related 5-HT may play a role in overweight/obesity-related EC.

Similarly, in this study, the BDNF levels of college students played a partial mediating role between overweight/obesity and EC. Our findings support the existing literature. El- Gharbawy et al., (2006) reported that overweight adolescents have lower BDNF levels and poor performance of EC than normal-weight adolescents [54]. In addition, overweight/obesity probably result in lower BDNF levels, which might lead to a lack of neurotrophic support in the adult brain and cause dysfunction of the hippocampus [66], presumably impairing performance of EC [67]. Regarding the level of BDNF in adolescents in this study, serum BDNF levels regulated the relationship between overweight/obesity and EC with an impact of 7.30%. Thus, both these results and ours provide preliminary evidence in support of the idea that overweight/obesity-related BDNF may play a role in overweight/obesity-related EC.

Our study extends prior research by providing the evidence that BDNF and 5-HT are potential biological mechanism underlying the association between obesity and EC. Nevertheless, this study was limited by several factors. Firstly, despite the study included a large sample size, it only covers the effects of overweight/obesity on serum 5-HT and BDNF levels as well as the EC response in young and fit healthy adults. Cognizant of this, further studies comprising of different populations such as patients with depressive disorders and more comprehensive methodological approaches such as downstream signaling and receptor expression are required to improve the relevance [68]. Secondly, future studies should include more detailed information about the fat mass, hydration status, and body composition of the participants by incorporating other analyses such as bio-impedance analysis and hematocrit [69]. This is because these factors also influence 5-HT and BDNF production, concentration and responsiveness. Thirdly, overweight/obesity did not account for fat distribution nor discriminate between lean body mass and fat mass despite it being the most commonly used measure of adiposity in previous epidemiological studies because of its feasibility and reliability [70]. Finally, our finding is consistent with the theories that overweight/obesity is harmful to the performance of EC. However, several studies have observed other associations between overweight/obesity and EC, generating speculation that dysregulation of EC could exacerbate poor decision-making with regard to diet and thus contribute to negative health outcomes, including excessive weight gain [71,72,73]. Future studies will also consider the intermediary role that 5-HT and BDNF may play between the EC and overweight/obesity.

## 5. Conclusions

In conclusion, using a sample drawn from a study of college students, we found that overweight/obesity was related to decreased 5-HT and BDNF, which was itself related to lower EC. We examined this potential role, and found that 5-HT and BDNF mediated the association between overweight/obesity and EC. Our results, therefore, could be taken to indicate that overweight/obesity-related 5-HT and BDNF may be one biological pathway underpinning links between overweight/obesity and EC.

## Figures and Tables

**Table 1:** General characteristics of participants based on gender (M ± SD).

	Male (*n* = 239)	Female (*n* = 210)	Total (*n* = 449)	*t*	*Cohen’ s d*
Age (year)	20.11 ± 6.28	19.41 ± 2.03	19.78 ± 4.79	−1.57	0.15
Height (cm)	172.78 ± 5.81	161.11 ± 5.17	167.32 ± 8.02	−22.36	2.12
Weight (kg)	68.77 ± 12.20	57.62 ± 9.56	63.56 ± 12.36	−10.84 *	1.01
BMI (kg/m^2^)	23.02 ± 3.79	22.21 ± 3.55	22.64 ± 3.70	−2.40	0.22
Serum 5-HT (ng/mL)	557.61 ± 139.05	535.93 ± 157.94	547.47 ± 148.41	−1.55	0.15
Serum BDNF (ng/mL)	6.29 ± 1.32	6.56 ± 1.48	6.414 ± 1.40	2.08	−0.20
Consistent ACC (%)	0.92 ± 0.13	0.91 ± 0.17	0.91 ± 0.15	−0.58	0.06
Consistent RT (ms)	504.49 ± 88.65	522.26 ± 101.43	215.80 ± 95.15	1.98	−0.19
Inconsistent ACC (%)	0.90 ± 0.15	0.89 ± 0.18	0.90 ± 0.16	−0.44	0.04
Inconsistent RT (ms)	503.07 ± 71.25	510.24 ± 74.80	506.43 ± 72.94	1.04	−0.10
Overweight	50	39	89		
Obesity	26	13	39		

A total sample size of *n* = 449 was included in the analysis. Descriptive data are presented as means (M) and standard deviations (SD). 5-HT: Serotonin, BDNF: Brain-derived Neurotrophic Factor, BMI: Body Mass Index, ACC: Accuracy, RT: Reaction Time. * Denotes statistical significance at *p* ≤ 0.05.

**Table 2:** Regression analysis of variable relations in the model.

		*R*	*R-sq*	*F*	*B*	*t*
Inconsistent RT	BMI	0.23	0.05	4.90	0.12 *	2.45
	5-HT				−0.10 *	−1.97
	BDNF				−0.11 *	−2.07
	Gender				0.13	1.32
	Years				−0.01	−0.68
5-HT	BMI	0.14	0.02	3.03	−0.119	−2.53
	Gender				−0.17	−1.78
	Years				0.00	0.47
BDNF	BMI	0.14	0.02	2.83	−0.09 *	−1.98
	Gender				0.18	1.88
	Years				0.00	0.41
Inconsistent RT	BMI	0.15	0.02	3.42	0.14 **	2.91
	Gender				0.12	1.30
	Years				−0.01	−0.76

All variables in the model are substituted into the regression equation by the standardized variables. 5-HT: Serotonin, BDNF: Brain-derived Neurotrophic Factor, BMI: Body Mass Index, RT: Reaction Time. * Denotes statistical significance at *p* ≤ 0.05. ** Denotes statistical significance at *p* ≤ 0.01.

**Table 3:** Mediating effect analysis.

		Effect	Boot SE	Boot LLCI	Boot ULCI	Proportion
Direct effect		0.12	0.05	0.02	0.21	83.94%
Indirect effect	5-HT	0.01	0.01	0.01 × 10^−2^	0.03	8.76%
	BDNF	0.01	0.01	0.07 × 10^−2^	0.02	7.30%
Total effect		0.14	0.05	0.04 × 10^−2^	0.23	100%

5-HT: Serotonin, BDNF: Brain-derived Neurotrophic Factor, SE: standard error, LLCI, ULCI: lower and upper levels for confidence interval. Boot SE, Boot LLCI, and Boot ULCI estimate the standard error of indirect effect, and the lower and upper limit of 95% confidence interval using the percentile bootstrap method with deviation correction, respectively.

## Data Availability

The data are not publicly available due to privacy or ethical reasons.

## References

[1] Collaborators G.B.D.O., Afshin A., Forouzanfar M.H., Reitsma M.B., Sur P., Estep K., Lee A., Marczak L., Mokdad A.H., Moradi-Lakeh M. (2017). Health effects of overweight and obesity in 195 countries over 25 years. N. Engl. J. Med..

[2] Hossain P., Kawar B., El Nahas M. (2007). Obesity and diabetes in the developing world—A growing challenge. N. Engl. J. Med..

[3] Xu J., Gao C. (2018). Physical activity guidelines for chinese children and adolescents: The next essential step. J. Sport Health Sci..

[4] Haslam D.W., James W.P.T. (2005). Obesity. Lancet.

[5] Ogden C.L., Yanovski S.Z., Carroll M.D., Flegal K.M. (2007). The epidemiology of obesity. Gastroenterology.

[6] Anderson A.S., Good D.J. (2017). Increased body weight affects academic performance in university students. Prev. Med. Rep..

[7] Ishihara T., Nakajima T., Yamatsu K., Okita K., Sagawa M., Morita N. (2020). Longitudinal relationship of favorable weight change to academic performance in children. Npj Sci. Learn..

[8] Naveed S., Lakka T., Haapala E.A. (2020). An overview on the associations between health behaviors and brain health in children and adolescents with special reference to diet quality. Int. J. Environ. Res. Public Health.

[9] Maranhao M.F., Estella N.M., Cury M.E., Amigo V.L., Picasso C.M., Berberian A., Campbell I., Schmidt U., Claudino A.M. (2015). The effects of repetitive transcranial magnetic stimulation in obese females with binge eating disorder: A protocol for a double-blinded, randomized, sham-controlled trial. BMC Psychiatry.

[10] Franz C.E., Xian H., Lew D., Hatton S.N., Puckett O., Whitsel N., Beck A., Dale A.M., Fang B., Fennema-Notestine C. (2019). Body mass trajectories and cortical thickness in middle-aged men: A 42-year longitudinal study starting in young adulthood. Neurobiol. Aging.

[11] Miyake A., Friedman N.P., Emerson M.J., Witzki A.H., Howerter A., Wager T.D. (2000). The unity and diversity of executive functions and their contributions to complex “frontal lobe” tasks: A latent variable analysis. Cogn. Psychol..

[12] Ware A.T., Kirkovski M., Lum J.A.G. (2020). Meta-analysis reveals a bilingual advantage that is dependent on task and age. Front. Psychol..

[13] Fleming C.B., Stevens A.L., Vivero M., Patwardhan I., Nelson T.D., Nelson J.M., James T.D., Espy K.A., Mason W.A. (2020). Executive control in early childhood as an antecedent of adolescent problem behaviors: A longitudinal study with performance-based measures of early childhood cognitive processes. J. Youth Adolesc..

[14] Lowe C.J., Reichelt A.C., Hall P.A. (2019). The prefrontal cortex and obesity: A health neuroscience perspective. Trends Cogn. Sci..

[15] He Q., Huang X., Zhang S., Turel O., Ma L., Bechara A. (2019). Dynamic causal modeling of insular, striatal, and prefrontal cortex activities during a food-specific go/nogo task. Biol. Psychiatry Cogn. Neurosci. Neuroimaging.

[16] Wen H.J., Tsai C.L. (2020). Neurocognitive inhibitory control ability performance and correlations with biochemical markers in obese women. Int. J. Environ. Res. Public Health.

[17] Kurhe Y., Mahesh R. (2017). Ondansetron ameliorates depression associated with obesity in high-fat diet fed experimental mice: An investigation-based on the behavioral, biochemical, and molecular approach. Indian J. Pharmacol..

[18] Beckman D., Santos L.E. (2013). The importance of serotonin in exercise-induced adult neurogenesis: New evidence from tph2-/- mice. J. Neurosci. Off. J. Soc. Neurosci..

[19] Brummelte S., Mc Glanaghy E., Bonnin A., Oberlander T.F. (2017). Developmental changes in serotonin signaling: Implications for early brain function, behavior and adaptation. Neuroscience.

[20] Dayan P., Huys Q.J. (2008). Serotonin, inhibition, and negative mood. PLoS Comput. Biol..

[21] Kovacic Petrovic Z., Nedic Erjavec G., Nikolac Perkovic M., Svob Strac D., Peraica T., Tudor L., Pivac N. (2019). The association between serotonin transporter polymorphism, platelet serotonin concentration and insomnia in non-depressed veterans with posttraumatic stress disorder. Psychiatr. Danub..

[22] Walley A.J., Asher J.E., Froguel P. (2009). The genetic contribution to non-syndromic human obesity. Nat. Rev. Genet..

[23] Speliotes E.K., Willer C.J., Berndt S.I., Monda K.L., Thorleifsson G., Jackson A.U., Lango Allen H., Lindgren C.M., Luan J., Magi R. (2010). Association analyses of 249,796 individuals reveal 18 new loci associated with body mass index. Nat. Genet..

[24] Marazziti D., Betti L., Baroni S., Palego L., Mucci F., Carpita B., Cremone I.M., Santini F., Fabbrini L., Pelosini C. (2020). The complex interactions among serotonin, insulin, leptin, and glycolipid metabolic parameters in human obesity. CNS Spectr..

[25] Pang J., Xi C., Huang X., Cui J., Gong H., Zhang T. (2016). Effects of excess energy intake on glucose and lipid metabolism in c57bl/6 mice. PLoS ONE.

[26] Labban R.S.M., Alfawaz H., Almnaizel A.T., Hassan W.M., Bhat R.S., Moubayed N.M., Bjorklund G., El-Ansary A. (2020). High-fat diet-induced obesity and impairment of brain neurotransmitter pool. Transl. Neurosci..

[27] Bar K.J., Kohler S., Cruz F., Schumann A., Zepf F.D., Wagner G. (2020). Functional consequences of acute tryptophan depletion on raphe nuclei connectivity and network organization in healthy women. NeuroImage.

[28] Drueke B., Schlaegel S.M., Seifert A., Moeller O., Grunder G., Gauggel S., Boecker M. (2013). The role of 5-ht in response inhibition and re-engagement. Eur. Neuropsychopharmacol. J. Eur. Coll. Neuropsychopharmacol..

[29] McAllister A.K., Katz L.C., Lo D.C. (1999). Neurotrophins and synaptic plasticity. Annu. Rev. Neurosci..

[30] Song M., Martinowich K., Lee F.S. (2017). Bdnf at the synapse: Why location matters. Mol. Psychiatry.

[31] Belviranli M., Okudan N., Kabak B., Erdogan M., Karanfilci M. (2016). The relationship between brain-derived neurotrophic factor, irisin and cognitive skills of endurance athletes. Physician Sportsmed..

[32] Coppell A.L., Pei Q., Zetterstrom T.S.C. (2003). Bi-phasic change in bdnf gene expression following antidepressant drug treatment. Neuropharmacology.

[33] Lee H.Y., Kim Y.K. (2008). Plasma brain-derived neurotrophic factor as a peripheral marker for the action mechanism of antidepressants. Neuropsychobiology.

[34] Cizza G., Rother K.I. (2012). The brain and obesity lectures series—The beginning of a new field?. Ann. N. Y. Acad. Sci..

[35] Lee S.S., Yoo J.H., Kang S., Woo J.H., Shin K.O., Kim K.B., Cho S.Y., Roh H.T., Kim Y.I. (2014). The effects of 12 weeks regular aerobic exercise on brain-derived neurotrophic factor and inflammatory factors in juvenile obesity and type 2 diabetes mellitus. J. Phys. Ther. Sci..

[36] Jo D., Son Y., Yoon G., Song J., Kim O.Y. (2020). Role of adiponectin and brain derived neurotrophic factor in metabolic regulation involved in adiposity and body fat browning. J. Clin. Med..

[37] Oh K.J., Lee D.S., Kim W.K., Han B.S., Lee S.C., Bae K.H. (2016). Metabolic adaptation in obesity and type ii diabetes: Myokines, adipokines and hepatokines. Int. J. Mol. Sci..

[38] Sweat V., Yates K.F., Migliaccio R., Convit A. (2017). Obese adolescents show reduced cognitive processing speed compared with healthy weight peers. Child. Obes..

[39] Mullins C.A., Gannaban R.B., Khan M.S., Shah H., Siddik M.A.B., Hegde V.K., Reddy P.H., Shin A.C. (2020). Neural underpinnings of obesity: The role of oxidative stress and inflammation in the brain. Antioxidants.

[40] Walsh J.J., D’Angiulli A., Cameron J.D., Sigal R.J., Kenny G.P., Holcik M., Doucette S., Alberga A.S., Prud’homme D., Hadjiyannakis S. (2018). Changes in the brain-derived neurotrophic factor are associated with improvements in diabetes risk factors after exercise training in adolescents with obesity: The hearty randomized controlled trial. Neural Plast..

[41] Chen C., Chen W., Chen C., Moyzis R., He Q., Lei X., Li J., Wang Y., Liu B., Xiu D. (2013). Genetic variations in the serotoninergic system contribute to body-mass index in chinese adolescents. PLoS ONE.

[42] Araki S., Yamamoto Y., Dobashi K., Asayama K., Kusuhara K. (2014). Decreased plasma levels of brain-derived neurotrophic factor and its relationship with obesity and birth weight in obese japanese children. Obes. Res. Clin. Pract..

[43] Aznar S., Hervig Mel S. (2016). The 5-ht2a serotonin receptor in executive function: Implications for neuropsychiatric and neurodegenerative diseases. Neurosci. Biobehav. Rev..

[44] Calderon J., Bellinger D.C. (2015). Executive function deficits in congenital heart disease: Why is intervention important?. Cardiol. Young.

[45] Ronan L., Alexander-Bloch A., Fletcher P.C. (2020). Childhood obesity, cortical structure, and executive function in healthy children. Cereb. Cortex.

[46] Groppe K., Elsner B. (2017). Executive function and weight status in children: A one-year longitudinal perspective. Child. Neuropsychol. J. Norm. Abnorm. Dev. Child. Adolesc..

[47] Kraus C., Baldinger P., Rami-Mark C., Gryglewski G., Kranz G.S., Haeusler D., Hahn A., Wadsak W., Mitterhauser M., Rujescu D. (2014). Exploring the impact of bdnf val66met genotype on serotonin transporter and serotonin-1a receptor binding. PLoS ONE.

[48] Du T., Sun X., Yin P., Huo R., Ni C., Yu X. (2013). Increasing trends in central obesity among chinese adults with normal body mass index, 1993–2009. BMC Public Health.

[49] Chen A.G., Yan J., Yin H.C., Pan C.Y., Chang Y.K. (2014). Effects of acute aerobic exercise on multiple aspects of executive function in preadolescent children. Psychol. Sport Exerc..

[50] WHO (2004). Appropriate body-mass index in asian populations and its implications for policy and intervention strategies. Lancet.

[51] Opel N., Redlich R., Kaehler C., Grotegerd D., Dohm K., Heindel W., Kugel H., Thalamuthu A., Koutsouleris N., Arolt V. (2017). Prefrontal gray matter volume mediates genetic risks for obesity. Mol. Psychiatry.

[52] Opel N., Martin S., Meinert S., Redlich R., Enneking V., Richter M., Goltermann J., Johnen A., Dannlowski U., Repple J. (2019). White matter microstructure mediates the association between physical fitness and cognition in healthy, young adults. Sci. Rep..

[53] Mackey S., Chaarani B., Kan K.J., Spechler P.A., Orr C., Banaschewski T., Barker G., Bokde A.L.W., Bromberg U., Buchel C. (2017). Brain regions related to impulsivity mediate the effects of early adversity on antisocial behavior. Biol. Psychiatry.

[54] El-Gharbawy A.H., Adler-Wailes D.C., Mirch M.C., Theim K.R., Ranzenhofer L., Tanofsky-Kraff M., Yanovski J.A. (2006). Serum brain-derived neurotrophic factor concentrations in lean and overweight children and adolescents. J. Clin. Endocr. Metab..

[55] Zimmer P., Stritt C., Bloch W., Schmidt F.P., Hubner S.T., Binnebossel S., Schenk A., Oberste M. (2016). The effects of different aerobic exercise intensities on serum serotonin concentrations and their association with stroop task performance: A randomized controlled trial. Eur. J. Appl. Physiol..

[56] Gallagher P., Massey A.E., Young A.H., McAllister-Williams R.H. (2003). Effects of acute tryptophan depletion on executive function in healthy male volunteers. BMC Psychiatry.

[57] Lee S.H., Son C., Yeo S., Ha I.H. (2019). Cross-sectional analysis of self-reported sedentary behaviors and chronic knee pain among south korean adults over 50 years of age in knhanes 2013–2015. BMC Public Health.

[58] Vakhrusheva J., Marino B., Stroup T.S., Kimhy D. (2016). Aerobic exercise in people with schizophrenia: Neural and neurocognitive benefits. Curr. Behav. Neurosci. Rep..

[59] Van Deutekom A.W., Chinapaw M.J., Vrijkotte T.G., Gemke R.J. (2013). Study protocol: The relation of birth weight and infant growth trajectories with physical fitness, physical activity and sedentary behavior at 8–9 years of age—The abcd study. BMC Pediatr..

[60] Tomlin D., Naylor P.J., McKay H., Zorzi A., Mitchell M., Panagiotopoulos C. (2012). The impact of action schools! Bc on the health of aboriginal children and youth living in rural and remote communities in british columbia. Int. J. Circumpolar Health.

[61] Button B.L.G., Martin G., Clark A.F., Graat M., Gilliland J.A. (2020). Examining factors of accelerometer-measured sedentary time in a sample of rural canadian children. Children.

[62] Masdeu J.C. (2011). Neuroimaging in psychiatric disorders. Neurother. J. Am. Soc. Exp. Neurother..

[63] Rahm C., Liberg B., Kristoffersen-Wiberg M., Aspelin P., Msghina M. (2014). Differential effects of single-dose escitalopram on cognitive and affective interference during stroop task. Front. Psychiatry.

[64] Montoya E.R., Terburg D., Bos P.A., van Honk J. (2012). Testosterone, cortisol, and serotonin as key regulators of social aggression: A review and theoretical perspective. Motiv. Emot..

[65] Jorgensen C., Wang Z. (2020). Hormonal regulation of mammalian adult neurogenesis: A multifaceted mechanism. Biomolecules.

[66] Seifert B., Eckenstaler R., Ronicke R., Leschik J., Lutz B., Reymann K., Lessmann V., Brigadski T. (2016). Amyloid-beta induced changes in vesicular transport of bdnf in hippocampal neurons. Neural Plast..

[67] Libman-Sokolowska M., Drozdowicz E., Nasierowski T. (2015). Bdnf as a biomarker in the course and treatment of schizophrenia. Psychiatr. Pol..

[68] Hodge S., Bunting B.P., Carr E., Strain J.J., Stewart-Knox B.J. (2012). Obesity, whole blood serotonin and sex differences in healthy volunteers. Obes. Facts.

[69] Jones D.H., Nestore M., Henophy S., Cousin J., Comtois A.S. (2014). Increased cardiovascular risk factors in breast cancer survivors identified by routine measurements of body composition, resting heart rate and arterial blood pressure. SpringerPlus.

[70] Vazquez G., Duval S., Jacobs D.R., Silventoinen K. (2007). Comparison of body mass index, waist circumference, and waist/hip ratio in predicting incident diabetes: A meta-analysis. Epidemiol. Rev..

[71] Smith E., Hay P., Campbell L., Trollor J.N. (2011). A review of the association between obesity and cognitive function across the lifespan: Implications for novel approaches to prevention and treatment. Obes. Rev. Off. J. Int. Assoc. Study Obes..

[72] Cournot M., Marquie J.C., Ansiau D., Martinaud C., Fonds H., Ferrieres J., Ruidavets J.B. (2006). Relation between body mass index and cognitive function in healthy middle-aged men and women. Neurology.

[73] Laurent J.S., Watts R., Adise S., Allgaier N., Chaarani B., Garavan H., Potter A., Mackey S. (2020). Associations among body mass index, cortical thickness, and executive function in children. JAMA Pediatr..

